# Orexin and pupillary modulation for hunger control

**DOI:** 10.1073/pnas.2619759123

**Published:** 2026-10-28

**Authors:** Seher Kosar, Eva Bracey, Nikola Grujic, Yihui Du, Daria Peleg-Raibstein, Denis Burdakov

**Affiliations:** ^a^ ETH Zürich, Department of Health Sciences and Technology, Neuroscience Center Zürich, Institute for Neuroscience, Schwerzenbach 8603, Switzerland

**Keywords:** hypothalamus, hunger, arousal, orexin, hypocretin

## Abstract

Fasting-based interventions improve metabolic health and are also explored as adjuncts in other clinical settings, but hunger remains a barrier to adherence. Fasting activates AgRP/NPY neurons (ANs), a hunger-promoting population whose inhibition reduces eating in hungry animals. Here we identify hypocretin/orexin neurons (HONs), classically associated with arousal, as rapid suppressors of AN activity. HON ablation increased AN population activity, whereas HON optostimulation rapidly suppressed it. Combined recordings of AN activity and pupil size—a direct arousal metric not previously linked to AN dynamics—revealed that HONs contributed to pupil dilation-associated AN suppression. In fasted mice, sensory stimulation rapidly recruited this pupil-HON pathway, and suppressed AN activity and eating. These findings reveal an arousal–hunger pathway through which self-initiated pupil dilations, or dilations driven by salient environmental events, interrupt fasting-induced hunger signals. They also suggest a framework for noninvasive, sensory-, and pupillometry-guided approaches to hunger control during fasting.

## Significance

This study reveals a genetically defined arousal-hunger circuit that enables mental engagement or external sensory input to naturally interrupt the urge to eat. These findings in mice suggest that noninvasive sensory tools, guided by monitoring pupil changes to optimize arousal, could provide a promising framework for hunger control during fasting and motivate future evaluation in humans.

Fasting imposes a powerful internal state that reshapes behavior, metabolism, and motivation. Fasting-based interventions can improve metabolic health in highly prevalent medical conditions such as obesity and diabetes (1–5), and are increasingly studied as adjuncts in clinical settings such as cancer therapy (6). Emerging work is also examining whether fasting-based interventions improve neuropsychiatric symptoms (7–9). Across all these contexts and regardless of exact fasting interventions, however, the aversiveness of hunger remains a major barrier to adherence and tolerability. Understanding how hunger can be rapidly suppressed is therefore important both for basic neuroscience and for the design of behavioral or sensory strategies that support fasting-based interventions.

Fasting creates a behavioral conflict. On the one hand, it triggers the aversive, food-focused state, driven by activation of the hypothalamic AgRP/NPY neurons (ANs) (10–12). On the other hand, survival requires hungry animals to be able to rapidly switch from this food-focused state to a higher arousal state (exploratory, nonfood focus), to process novel sensory stimuli in order to detect danger and react to it. This conflict of engaging vs. disengaging in eating behavior could be resolved if increased arousal suppressed ANs. Such a mechanism would be translationally important because arousal is easily modified and can even be self-regulated in humans (13); however, its existence remains an open question. For instance, transmitters released by the hypocretin/orexin neurons (HONs)—key correlates of both evoked and spontaneous arousals (14–16)—are currently known only to activate ANs (17, 18). While some studies show that stressful events might suppress ANs (19–21), they lack direct measures of arousal, such as pupil size tracking (22, 23). Effects on ANs of isolated arousal variables—such as spontaneous pupil dilations or HON firing—remain unknown.

To resolve these gaps in knowledge, here we combined AN activity recordings with HON-specific manipulations and direct, high temporal resolution quantification of arousal using video pupillometry. We found that HON ablation increases net AN population activity, while HON optostimulation suppresses it, through a previously overlooked indirect pathway. In fasted mice, we found that pupil dilations coincided with rapid, HON-dependent suppressions of the AN activity, and pupil-dilating stimuli evoked HON-dependent reduction of eating. These results indicate that HONs are recruited concurrently with pupil-linked arousal to implement a rapidly acting “arousal brake” on hunger in the brain.

## Results

### AN Activity Is Increased After HON Ablation and Reduced During Targeted HON Stimulation.

**Fig. 1.**
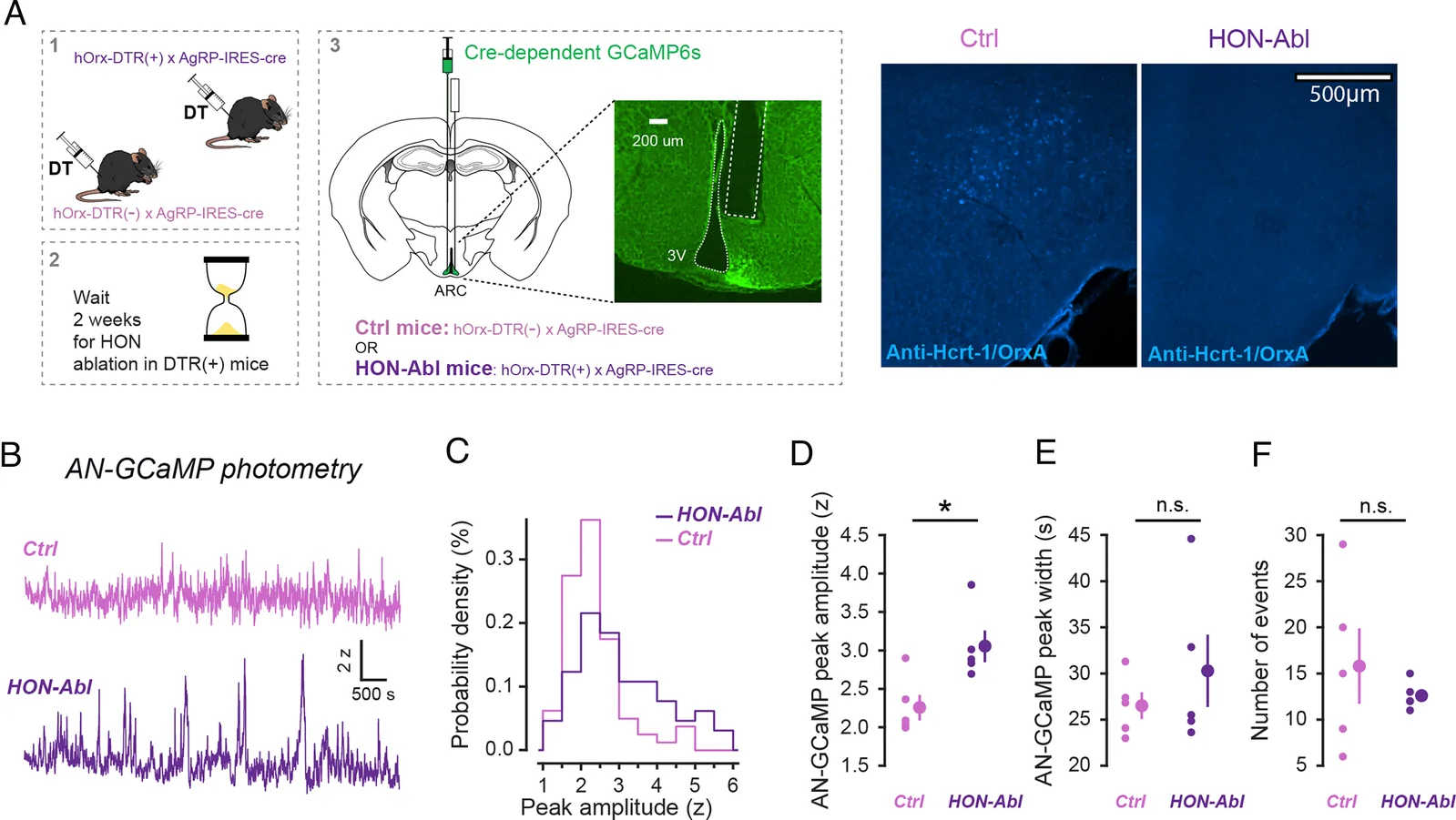
HON ablation increases spontaneous AN activity in freely-moving, ad lib fed mice. (*A*) *Left*, Schematic for the HON-ablation protocol (steps 1 to 2) and fiber photometry recording of AN in HON-ablated mice (HON-Abl) and control mice (Ctrl) (step 3). *Right*, Representative orexin immunostaining in a Ctrl mouse (*Left*) and a HON-ablated mouse. (*B*) Example recordings of spontaneous AN activity in Ctrl and HON-ablated mice (representative examples of n = 5 mice in each group). (*C*) Amplitude distributions of AN activity spikes, showing a shift to larger amplitudes in HON-ablated mice (one-sided Kolmogorov–Smirnov test; ***P = 0.000089, n = 5 mice in each group). (*D*–*F*) Quantifications of the AN spikes: peak amplitude (*D*), width (*E*), and number (*F*) (unpaired two-tailed test; *P = 0.02 in *D*; ns, *P* = 0.39, 0.46 for *E* and *F*, respectively, n = 5 per each group).

To establish how endogenous HONs influence ANs, we first selectively ablated HONs in adult mice and examined the resulting changes in spontaneous AN activity. We performed this experiment in ad libitum-fed mice to determine whether endogenous HONs constrain AN activity even outside the maximally hunger-activated fasted state. To achieve this, we crossed two previously validated transgenic mouse models: hOrx-DTR mice (24) to enable HON ablation after injection of DT (24–26), and AgRP-IRES-cre mice (27) to enable expression of cre-dependent activity sensor GCaMP in ANs (Fig. 1*A*). We injected DT into adult control and DTR mice, producing complete HON ablation in the latter after 2 wk, as previously reported (24) (Fig. 1*A*). We expressed GCaMP in ANs by injecting AAV carrying cre-dependent GCaMP6s into the arcuate hypothalamus, and recorded spontaneous activations of AN population activity (28) using fiber photometry in freely moving mice in a neutral arena.

Based on the reports of direct excitatory effects of hypocretin/orexin on ANs (17, 18), we expected that the HON ablation would be associated with reduced AN activity. Instead, we found that HON ablation increased amplitudes of spontaneous AN activations (Fig. 1 *B*–*F*, statistics are provided in the figure legends). This reveals that, even in the fed state, removal of HONs disinhibited the spontaneous AN activity, suggesting that endogenous HON tone normally inhibits it.

**Fig. 2.**
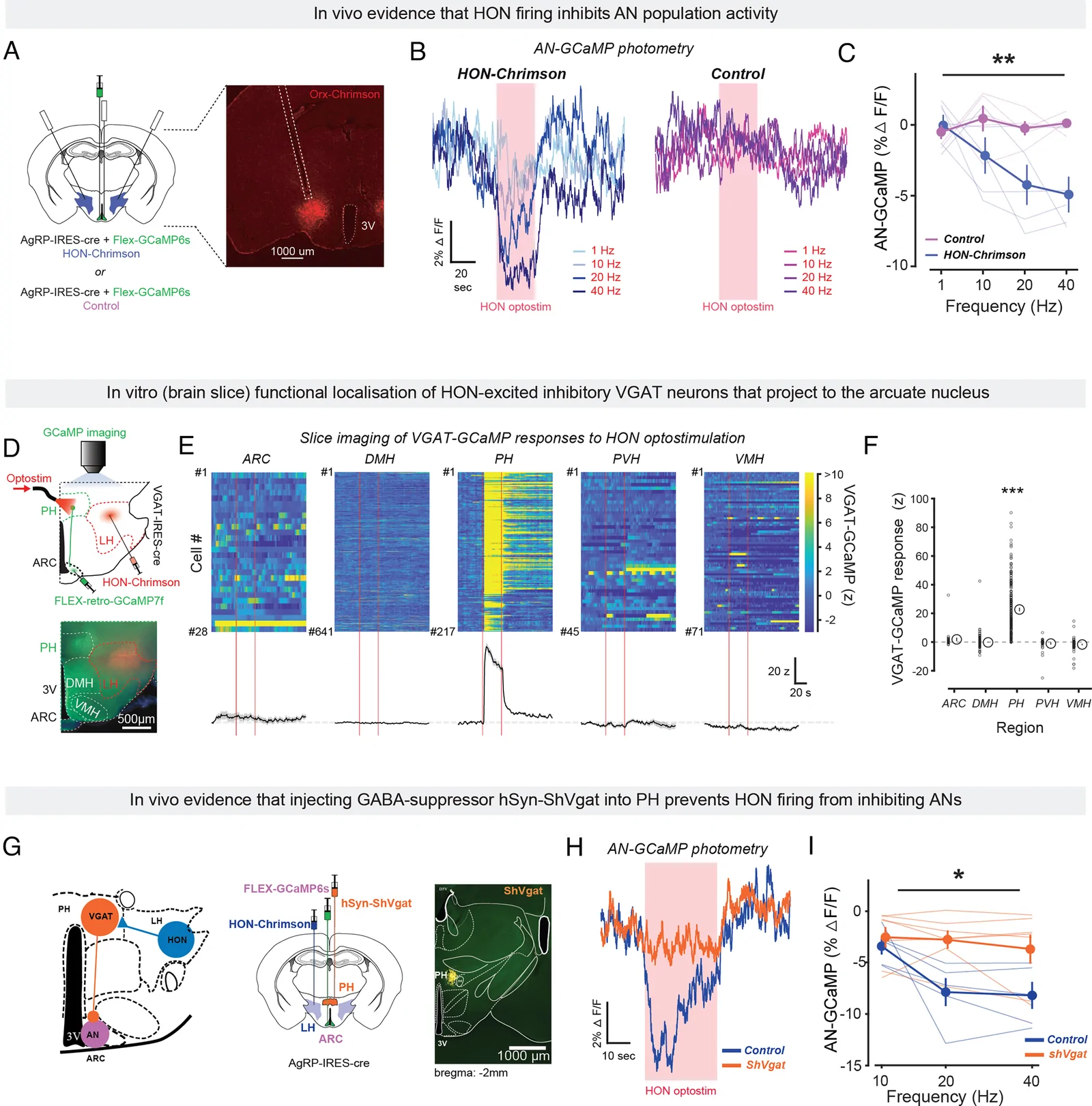
HON optostimulation inhibits AN population activity via an intrahypothalamic GABAergic relay. (*A*) *Left*, schematic of stimulated (HON) and recorded (AN) neurons. *Right*, examples of corresponding histological verification of expression and fiber placements. (*B*) AN-GCaMP6s responses to HON optostimulation (red bar) at different frequencies (means of n = 5 mice in each group). (*C*) Quantification of (*B*) (Two-way RM ANOVA; control/chrimson: F_(1, 8)_ = 13.27, **P = 0.0066; n = 5 mice in each group). Post hoc analysis (HON-Chrimson vs. Control): 1 Hz (*P* = 0.617), 10 Hz (*P* = 0.134), 20 Hz (*P = 0.029), 40 Hz (**P = 0.005). (*D*) *Top*, Experimental design for single-cell calcium imaging of ARC-projecting VGAT-GCaMP7f neuron responses to stimulation of HON axons in various hypothalamic nuclei (ARC, DMH, PH, PVH, VMH). *Bottom*, Histology shows an example slice where the posterior hypothalamic nucleus (PH) responded to HON optical stimulation. (*E*) *Top*, Heatmaps displaying responses of ARC-projecting VGAT-GCaMP7f neurons to HON Chrimson-expressing axon stimulation in different nuclei. The onset and offset of 635 nm laser stimulation are indicated by red lines. (*F*) Quantification of population shown in (*E*) (one-sample one-tailed *t* test; ***P < 0.0001, n = 217 cells). (*G*) *Left*, the proposed feedforward inhibitory circuit: LH^HON^ -> PH^VGAT^ -> ARC^AGRP^. *Middle*, experimental design for functional circuit experiment, selective knockdown of VGAT in PH, and simultaneous HON optostimulation and AN recording. *Right*, example image showing viral expression of ShVgat. (*H*) Averaged temporal dynamics of AN-GCaMP6s responses to HON optostimulation (laser bar) at 20 Hz in control (means of n = 5 mice) and ShVgat (VGAT knockdown in PH) mice (means of n = 7 mice). (*I*) Quantification of (*H*) (Two-way RM ANOVA; control vs ShVgat: F_(1, 10)_ = 5.913, *P = 0.0353; n = 5 control mice, n = 7 ShVgat mice). Post hoc analysis (ShVgat vs. control): 10 Hz (*P* = 0.603), 20 Hz (**P = 0.004), 40 Hz (*P = 0.011).

To complement this chronic loss-of-function experiment, we next acutely stimulated HONs while recording AN activity. To achieve this, in the LH of AgRP-IRES-cre mice, we injected an AAV previously confirmed to selectively express the excitatory opsin Chrimson in mouse HONs [orx-Chrimson (26, 29)], and also expressed the cre-dependent GCaMP sensor in ANs (Fig. 2*A*). Optostimulating HON-Chrimson neurons through LH-implanted fibers with different laser-pulse frequencies caused rapid, frequency-dependent suppression of AN-GCaMP signals, in HON-Chrimson mice but not in control mice without the opsin (Fig. 2 *B* and *C*). We reasoned that HON→AN inhibition likely occurs through an intermediate inhibitory relay, since ANs are not responsive to the HON’s inhibitory transmitter dynorphin, and other HON transmitters are excitatory (30–33). Indeed, it was largely abolished when we used established knockdown tools (34) to disrupt inhibitory GABAergic output from one of the AN-innervating hypothalamic regions, PH (35), where we observed a high density of HON-activated, arcuate-projecting GABAergic neurons (Fig. 2 *D*–*I*, see *Methods*).

Together with the ablation results, this confirms that HONs exert inhibitory control over AN population activity in vivo.

### HONs Contribute to AN Suppression During Spontaneous Pupil Dilations.

In humans and mice, pupil size spontaneously fluctuates even under constant light; discovering neural correlates of these fluctuations is of high translational interest for developing new treatments and diagnostics (13, 22, 23).

**Fig. 3.**
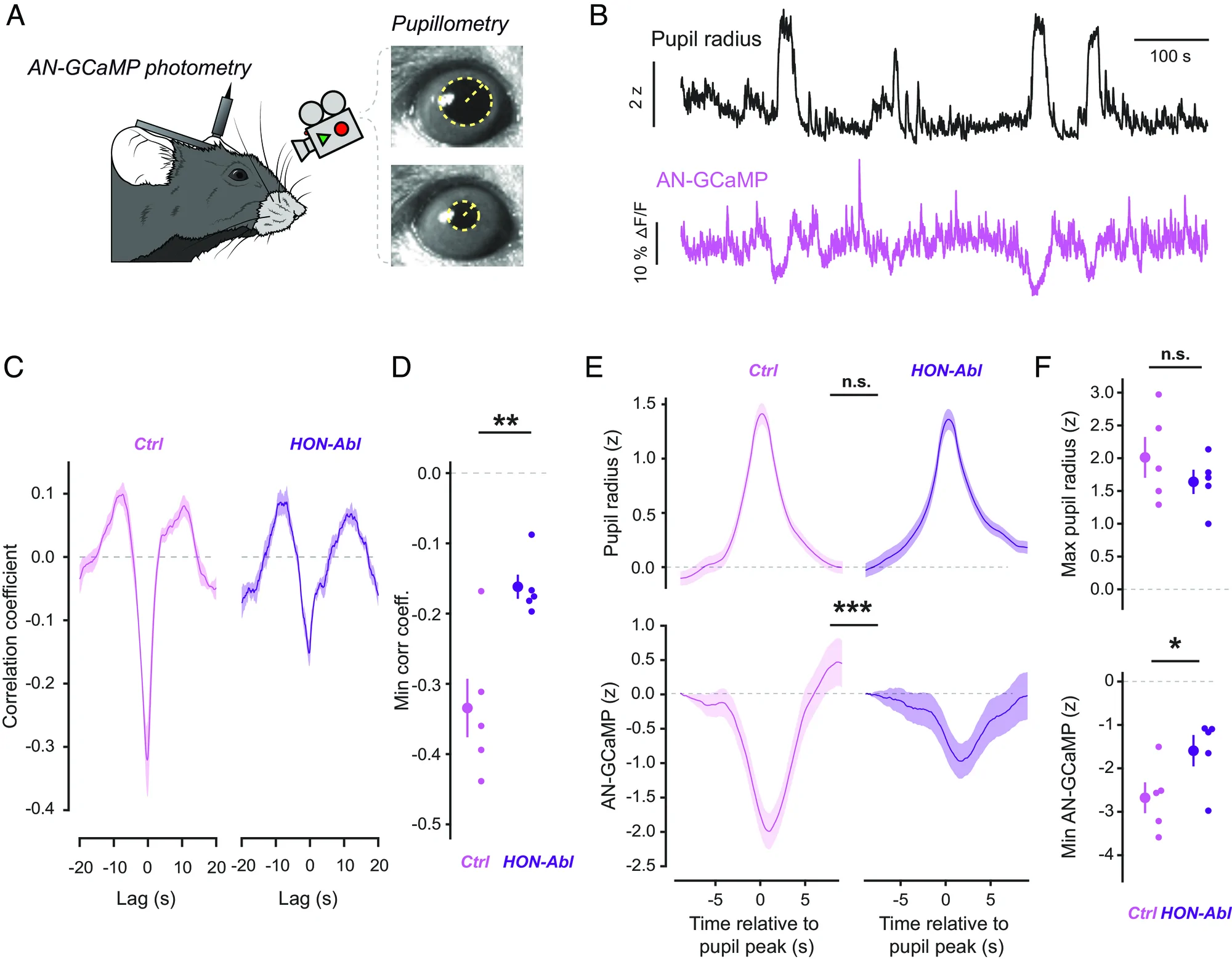
Suppression of AN activity during spontaneous pupil dilations in fasted mice, and the role of HONs in this phenomenon. (*A*) Method for simultaneous recording of pupil size and AN activity. (*B*) Example recording of pupil radius (*Top*, black) and AN-GCaMP6s activity (*Bottom*, pink) (representative trace of n = 5 mice). (*C*) Cross-correlation analysis of pupil radius and AN-GCaMP6s signals in HON-ablated (HON-Abl, purple) and control (Ctrl, pink) mice (n = 5 mice per group). (*D*) Quantification of correlation minima from (*C*) (unpaired one-tailed *t* test, **P = 0.0086, n = 5 mice in each group). (*E*) Pupil dilation events of similar magnitude (*Top*) aligned with corresponding AN-GCaMP6s signals (*Bottom*) in HON-ablated and control mice. (one-tailed *t* test, ns, *P* = 0.59 for pupil radius; ***P < 0.001 for AN-GCaMP; 196 events from n = 5 Ctrl mice, 172 events from n = 5 HON-ablated mice; comparison of means of −5 to +5 s epochs). (*F*) Quantification of data in (*E*). *Top*: Maximum pupil radius (unpaired one-tailed *t* test; ns, *P* = 0.166); *Bottom*: Minimum AN-GCaMP (unpaired one-tailed *t* test; *P = 0.032) (n = 5 mice per group).

Having established that HON activation suppresses ANs, and given that HON activity is positively correlated with spontaneous pupil dilations (15), we hypothesized that the dilations would negatively correlate with AN activity, and this correlation would be HON-dependent, in fasted mice. To test this, we concurrently recorded AN-GCaMP signals and pupil size (Fig. 3*A*), and indeed found AN activity and pupil size to be negatively correlated (Fig. 3 *B*–*F*). This establishes that moment-to-moment fluctuations in arousal state, as indexed by pupil size, are negatively coupled to AN activity during fasting. In the HON-ablated mice, this negative correlation between pupil size and AN activity was significantly reduced (Fig. 3 *C*–*F*).

Together, these results identify endogenous HONs as contributors to the suppression of AN population activity during internally-driven, spontaneous pupil dilations in fasted mice.

### HONs Contribute to AN and Eating Suppression During Externally-Evoked Pupil Dilations.

Having shown that HONs contribute to AN suppression during spontaneous arousal fluctuations, we next asked whether HON-dependent suppression of ANs is also engaged by specific external stimuli. Food inhibits ANs in different ways: initial anticipatory inhibition by food cues, which is fast but transient if food is then not consumed; and slower but more sustained inhibition by gut nutrients and restoration of energy balance (10, 28, 36–38). We first presented mice with cues indicative of familiar, highly palatable foods (HPF, see *Methods*), which are well established to evoke the rapid anticipatory inhibition of ANs (10, 28, 36–39).

**Fig. 4.**
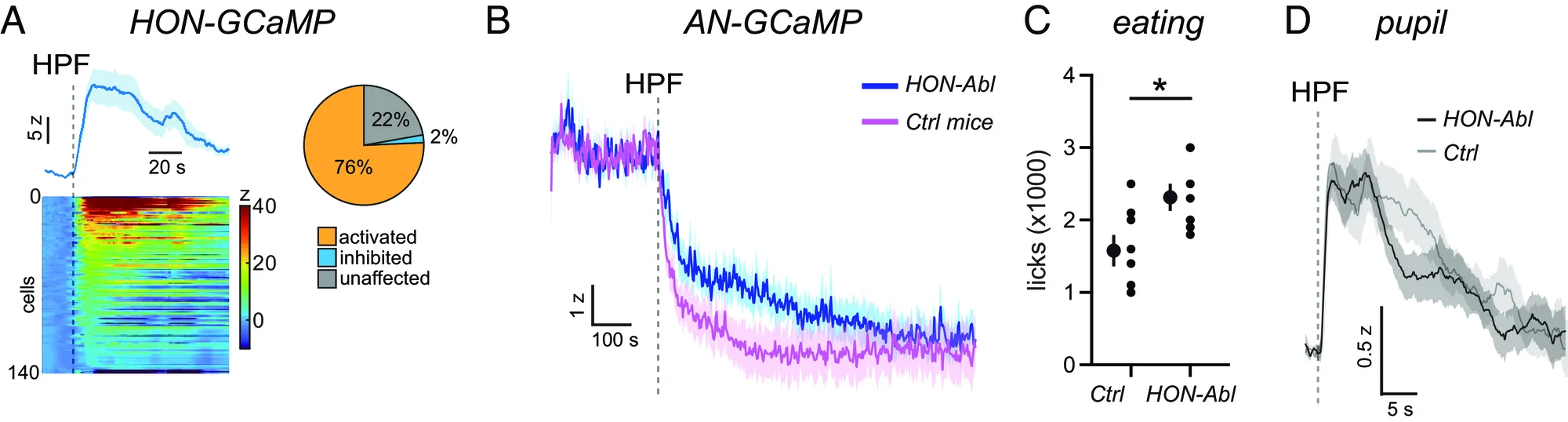
Involvement of HONs in neurobehavioral and arousal responses to food in fasted mice. (*A*) HON activity responses to HPF presentation (see *Methods*). Top trace shows population response, heatmap shows individual cells from the same time interval for n = 6 mice (140 cells, n = 20 to 25 cells per mouse). Pie chart summarizes the percentages of cells that were activated (76%, response: z > 4), inhibited (2%, response: z < 4), or unaffected (22%, response: −4 ≤ z ≤ 4). (*B*) AN-GCaMP6s responses to HPF presentation in HON-ablated (HON-Abl, blue) and control (Ctrl, magenta) mice. Two-way RM ANOVA, interaction: F_(7,126)_ = 2.62, *P* = 0.015; Bonferroni post hoc tests comparing Ctrl vs. HON-Abl mice in consecutive 100 s time bins starting at HPF: *P* < 0.05 for the first bin, *P* > 0.05 for subsequent bins; n = 9 HON-Abl and n = 11 Ctrl mice. (*C*) Licking behavior (total licks) during the 20-min period following milkshake presentation in Ctrl and HON-ablated mice (unpaired two-tailed *t* test; *P = 0.015, n = 8 mice in each group). (*D*) Temporal dynamics of the pupil response (z-score) in control and HON-ablated mice upon presentation of highly palatable food (HPF), n = 11 HON-Abl and 14 Ctrl mice (peak response comparison with unpaired two-tailed *t* test, *P* > 0.05).

Of note, the HPF cues also dilate pupils, and rapidly activate HONs (24, 40) (Fig. 4*A*). The purpose of this HON activation is unknown, but mimicking it with HON-specific optogenetic stimulation is sufficient to acutely reduce food intake [see figure 4 in (25)]. Based on this, we hypothesized that HONs contribute to the AN suppression by HPF cues. Consistent with this, we found that the initial rapid phase of AN suppression by HPF presentation is reduced in HON-ablated mice (Fig. 4*B*). Interestingly, the HON-ablated mice also subsequently consumed more HPF (Fig. 4*C*). Importantly, HPF-evoked pupil dilation amplitude was not substantially altered by HON-ablation in this experiment (Fig. 4*D*), demonstrating that the arousal response (pupil dilation) and AN inhibition are dissociable, and that HONs contribute to the latter.

**Fig. 5.**
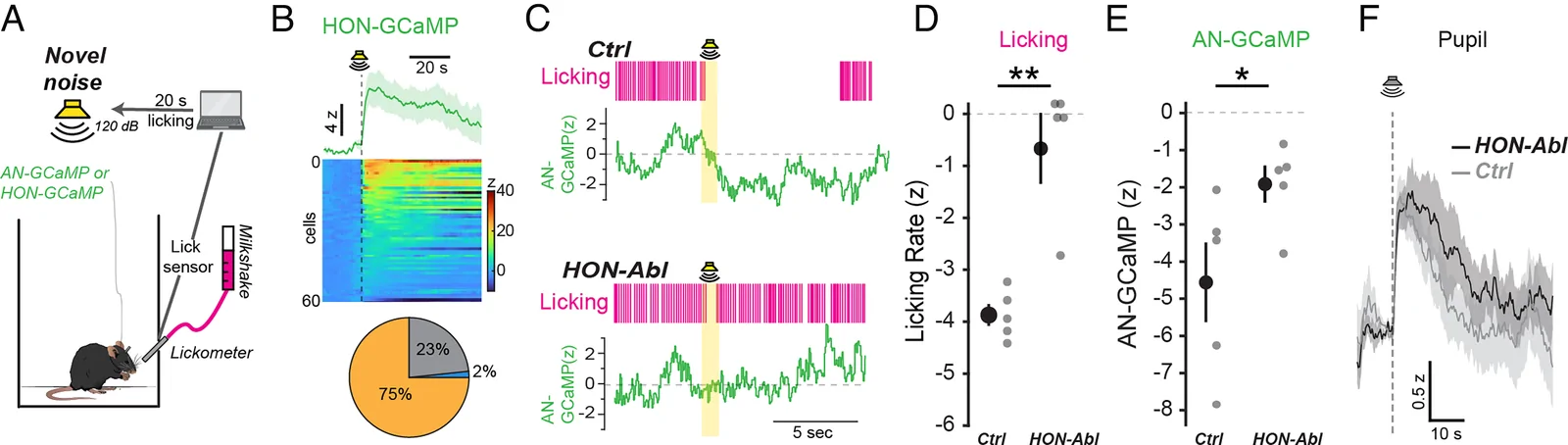
Role of HONs in effects of nonfood-evoked pupil dilations on hunger metrics in fasted mice. (*A*) Schematic of the “sudden noise” experiment. After 20 s of continuous licking of milkshake, a 1 s novel noise was presented (see *Methods*). (*B*) HON activity response to novel sudden noise (vertical dashed line), n = 6 mice. Top trace shows population response, and heatmap shows individual cells from the same time interval for n = 6 mice (60 cells, n = 5 to 15 cells per mouse). Pie chart summarizes the percentages of cells that were activated (75%), inhibited (2%), or unaffected (23%); responses classified using the same criteria as in Fig. 4*A*. (*C*) Example effects of the noise on licking behavior (pink raster) and AN-GCaMP6s activity (green trace) in Ctrl (*Top*) and HON-Abl (*Bottom*) mice. The yellow shaded area indicates the noise presentation (representative trace of Control n = 5 and HON-Abl n = 5 mice). (*D*) Quantification of data for licking rate (z-score) following the noise, Ctrl n = 5, HON-Abl n = 5 mice (unpaired two-tailed *t* test; **P = 0.0017). (*E*) Quantification of the AN-GCaMP6s response to sudden noise, n = 5 mice per group (unpaired one-tailed *t* test; *P = 0.0279). (*F*) Pupil size response (z-score) following startling sound, n = 11 HON-Abl and 14 Ctrl mice (peak response comparison by the unpaired two-tailed *t* test, *P* > 0.05).

Finally, we asked whether the HON-dependent suppression of ANs and eating can also be recruited by simple nonfood arousing stimuli, which might be of greater translational value for hunger suppression than food cues. To assess this, we briefly exposed fasted mice to a startling sound not previously heard by the mouse, and not associated with food, to dissociate arousal-linked AN suppression from food-cue processing (Fig. 5*A*). This stimulus potently activated HONs (Fig. 5*B*), which is in theory sufficient to acutely reduce food intake [figure 4 in (25)]. In control mice, the novel sound rapidly suppressed AN activity and reduced HPF intake, but in HON-ablated mice these responses were substantially reduced (Fig. 5 *C*–*E*). In contrast, the pupil response amplitudes were not substantially different in control and HON-ablated mice (Fig. 5*F*), reinforcing the dissociation between the arousal response itself and HON-dependent feeding suppression.

**Fig. 6.**
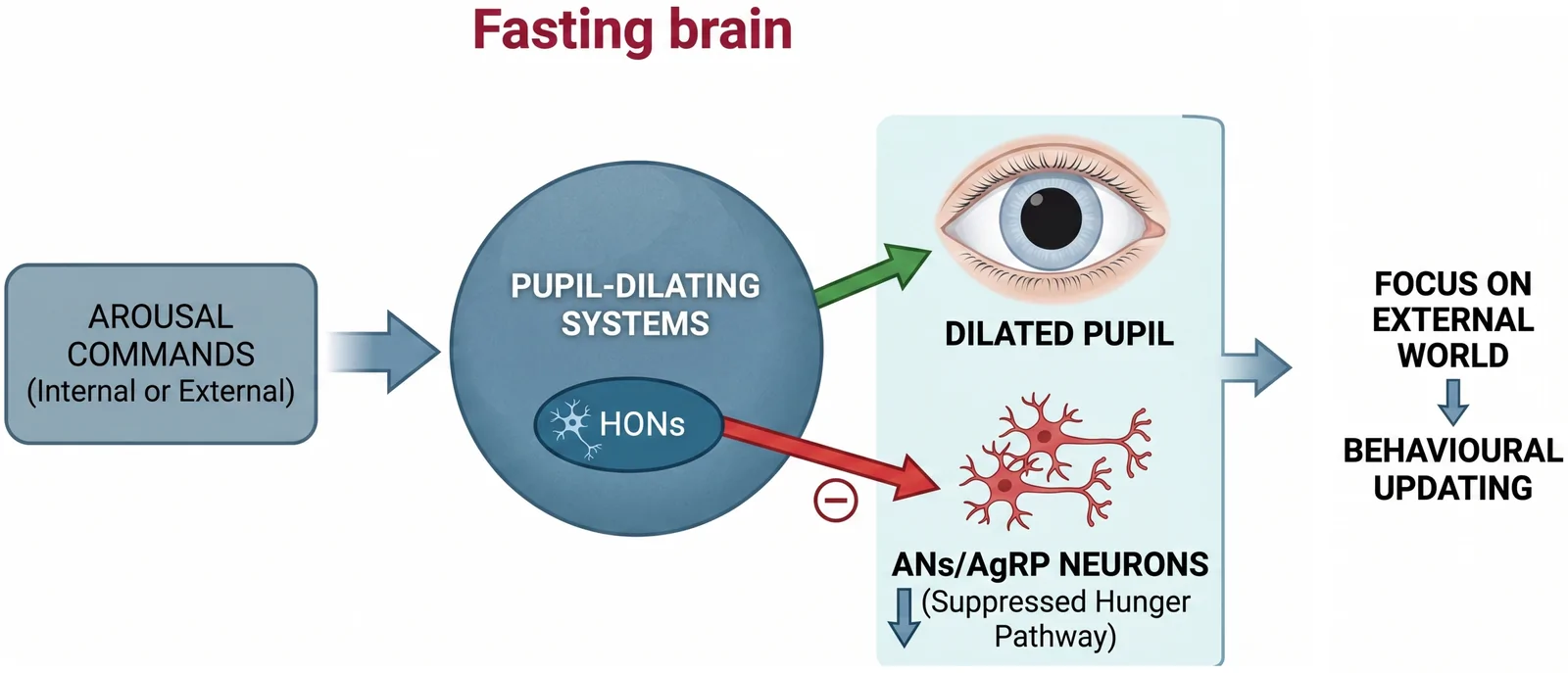
Orexin and pupillary modulation for hunger control. Our model for how and why HONs implement arousal-hunger orchestration. During fasting, AN activity promotes hunger and feeding. Internal or external arousal commands recruit multiple arousal systems, including pupil-dilating systems and HONs (23). HONs inhibit ANs (through an indirect GABA relay), alongside concurrent pupil dilation and hunger suppression, that may help animals explore the broader meaning of the environment and respond to opportunities or threats (see *Discussion*).

Overall, these results indicate that evoked pupil dilations coincide with HON-dependent suppressions of feeding and ANs. In this context, HONs do not appear to be the primary determinants of dilations themselves, but rather implement dilation-associated hunger suppression (Fig. 6).

## Discussion

*“There is the Great Hunger and there is the Little Hunger. The Little Hunger wants food for the belly; but the Great Hunger, the greatest hunger of all, is the hunger for meaning”* (41)

Hunger can suppress other motivational and behavioral states (20, 42–44), but animals must maintain flexible arousal levels to survive. Classic observations indicate that arousal also suppresses ongoing behaviors, which shifts focus to exploring the meaning of a situation, in terms of opportunities or threats (45–48). Our results indicate that, in fasted mice, during phasic pupil dilations hunger is rapidly suppressed via a specific class of arousal-coactivated cells, the HONs, that inhibit “hunger” neurons, the ANs. Importantly, in our experiments, pupil dilations could still occur without HONs, but they no longer effectively suppressed feeding and ANs. This suggests a revised cellular model for arbitration between arousal and hunger, wherein HONs implement rapid suppression of feeding and AN population activity during states of high arousal (Fig. 6).

Can our findings be reconciled with previous work? Suppression of hunger to learn the meaning of an unexpected arousing event—what Pavlov called “*Shto takoye?”* (what is it?) response—makes clear evolutionary sense (45). It can allow animals to assess novel environmental events without being dominated by hunger drive. By contrast, the rapid, preingestive suppression of AN activity by food discovery [reported in (10, 28, 36) and reproduced in Fig. 4*B*] may seem paradoxical, since animals generally eat the food they discover. However, we think it is also logical for survival. In our model of hunger–arousal interaction (Fig. 6), one possibility is that it may limit excessive focus on food. This “less eating, more vigilance” response to food discovery may be adaptive because food discovery is not only a nutritive event, but also a salient environmental event. In natural foraging contexts, feeding must often be balanced against environmental monitoring, competition, and threat. Thus, the transient, HON-modulated suppression of AN activity by food cues may contribute to maintaining general vigilance at the moment of food discovery, and/or have other roles including cephalic responses and learning (39). Our observation of the indirect HON→AN inhibition does not dispute the existence of direct HON→AN excitation reported or proposed by others (17, 18, 49), which we also observed in a fraction of ANs in our own in vitro studies [figure 2 *A*–*C* in (50)]. In terms of previous work on eating-related HON control, the preingestive HON-dependent suppression of ANs is conceptually compatible with the inhibition of HONs by the act of eating itself (24), that according to the present findings may maintain some degree of AN-driven hunger until sufficient calories are ingested. Finally, more broadly, the present findings are compatible with—and provide a neural rationale for—several known conditions where arousal and hunger change in opposite directions. These include anorexia nervosa [increased arousal, reduced eating (51, 52)], aging [increased arousal, reduced appetite, HON hyperactivity (53–56)], and orexin-deficient narcolepsy [reduced arousal and increased appetite (57–59)].

Our findings suggest further important directions for future work. There are indications that pupil size responses to food cues are smaller in overweight individuals (60, 61). It would be interesting to investigate whether this means that failure of the “arousal brake” on hunger could be a cause of obesity. Interestingly, recent work indicates that humans can be trained to self-regulate their pupil dilation via pupillometry-assisted biofeedback (13): it would be important to determine if this could be used for self-regulation of hunger. Exploring the dilation-associated AN inhibition as an anti-hunger measure in fasting therapies would also require greater knowledge about the role of ANs in some beneficial effects of fasting (21, 62–65). If ANs mediate some of these beneficial effects, in some fasting treatments, their inhibition may not be appropriate. On a cellular level, both the indirect inhibitory HON→AN pathway and the direct excitatory HON→AN pathway likely operate in the brain. The relative contributions of the direct excitatory and indirect inhibitory HON→AN pathways likely depend on brain state, neuromodulatory context, and the specific AN neuron subpopulation engaged, questions that represent important directions for future work. Specifically, the assumption that ANs are turned off by food cues in order to prevent overeating remains hypothetical, and another reason for this phenomenon could be that ANs mediate multiple food-seeking behaviors, and once the food is identified most of these behaviors (except eating) need to be suppressed. Indeed, while the majority of ANs are inhibited by food cues, a small subset is activated (10, 36), and it is tempting to speculate that this subset might correspond to the HON-activated ANs directly innervated by HONs (49). Finally, our finding that HON-dependent pathways depress both eating and ANs during arousal does not prove that the eating suppression is causally linked to PH GABAergic activation and AN suppression: it remains to be investigated what further HON outputs contribute to the observed feeding phenotype. Since sensory inhibition of AN was only partly abolished in the HON-deficient mice (Figs. 4*B* and 5*E*), other pathways for feedforward suppression of ANs and hunger also likely exist and could be explored as anti-hunger therapies (66–68). The roles of transient HON signals vs. tonic HON activity in modulatory and permissive control of hunger state also remain to be investigated; this would require temporally controlled optogenetic silencing of HONs combined with electrical recordings of HON and AN firing.

In summary, our findings suggest that physiological arousal attenuates fasting-induced hunger through a HON-dependent mechanism, enabling hungry animals to interrupt feeding when salient environmental events require behavioral updating. These insights may inform the development of noninvasive, sensory-, and pupillometry-based interventions for appetite management during periods of fasting.

## Methods

### Animal Procedures.

All procedures followed Swiss Federal Food Safety and Veterinary Office Welfare Ordinance (TSchV 455.1, approved by the Zurich Cantonal Veterinary Office). Adult (older than 22 wk) female and male C57BL/6 mice were used. Animals were housed in a 12-h reversed light–dark cycle at 22 °C with 55% humidity and had ad libitum access to standard chow (3430 Kliba Nafag, Kaiseraugst, Switzerland) and water unless stated otherwise. AgRP-IRES-cre mice and VGAT-IRES-cre mice were from Jackson Lab (Jax no. 012899 and 028862, respectively). To study mice without HONs, we selectively ablated HONs using the previously validated hOrx-DTR mouse model (24). To study AN activity in HON-ablated mice, AgRP-IRES-cre and hOrx-DTR mice were crossed (AgRP-IRES-Cre × hOrx-DTR mice). Experiments were performed during the active (dark) phase of the light cycle. Before the experiments, mice were either fed ad lib (Figs. 1 and 2), or fasted for 24 h (Figs. 3–5).

### Stereotaxic Surgeries and Viral Vectors.

Mice were anesthetized with 5% isoflurane, then transferred to the stereotaxic setup (Kopf Instruments, Tujunga, United States) and maintained on 1.5 to 2% isoflurane. They were given analgesic 30 min before anesthesia, and for 3 d post surgery. Lidocaine was applied to the scalp before an incision was made to access the cranium. Craniotomies were performed using a stereotax-mounted drill (RWD Life Sciences). Viral vectors and implants were positioned at specific coordinates (given below, as mm from Bregma unless stated otherwise). A Nanoject III injector was used to inject the viral vectors at a speed of 1 nl s^−1^.

For AN recordings, AAV9.CAG.Flex.GCaMP6s.WPRE.SV40 (titer ≥ 1.9 × 10^13^ vg/mL, from Addgene, MA, 100842-AAV9) was injected unilaterally into the arcuate nucleus of AgRP-IRES-cre, or AgRP-IRES-cre × hOrx-DTR mice, at the following coordinates: anteroposterior −1.75, mediolateral −0.28, dorsoventral −6.1, −6.0 to −5.9 (100 nl per site). Optic fibers (400 µm, 0.39 NA, 1.25 mm metal ferrule, Doric) were implanted at dorsoventral −5.85.

For HON recordings, AAV1.hORX.GCaMP6s.hGH (2.5 × 10^12^ GC ml^−1^, Vigene Biosciences), previously demonstrated to selectively target HONs (24), was injected at coordinates: anteroposterior −1.35, mediolateral −/+ 0.9, dorsoventral −5.7, −5.4, and −5.1 (50 nl per site). A GRIN lens (ProView™ Integrated Lens 0.6 mm × 7.3 mm, Inscopix) was then implanted at dorsoventral −5.1.

For HON optostimulation, injection of AAV9-hORX-ChrimsonR-mCherry (2 × 10^12^ GC ml^−1^, UZH Viral Vector Facility) previously demonstrated to selectively target HONs (29), or control injection (PBS), was performed bilaterally at: anteroposterior −1.45, mediolateral ±0.9, dorsoventral −4.7 and −4.8 (150 nl per site). For in vivo optostimulation of HONs, optic fibers (200-μm diameter, 0.39 NA, 1.25-mm metal ferrule; Thorlabs), angled 20-degrees from vertical, were placed 100 μm above injection sites.

For in vitro optogenetic-assisted calcium imaging probing functional connectivity from HON to arcuate-projecting VGAT neurons, we injected AAV9-hORX-ChrimsonR-mCherry into the lateral hypothalamus (coordinates as above) and AAVretro-Syn-FLEX-GCaMP7f (Addgene, at titer ≥7 × 10^12^ vg/mL, catalog# 104492) into the arcuate nucleus (coordinates as above) of VGAT-IRES-cre mice.

For selective knockdown of VGAT expression, we PH-injected VGAT-selective shRNA constructs (34) or PBS as control. The pAAV-hsyn-flex-dsRed-shVgat plasmid (Addgene, Plasmid #67845) was modified and packaged by Charles River as AAV1-hsyn-dsRed-shVgat (titer: 1 × 10^13^ GC/mL). This virus (1:5 dilution) or PBS was injected (150 nl per site) into PH (anteroposterior: −2.0, mediolateral: ±0.37, dorsoventral: −4.9) of AgRP-IRES-cre mice. In the same mice, Cre-dependent GCaMP6s and hOrx-ChrimsonR viruses were injected into ARC and LH respectively, and fibers were placed as described above.

For pupillometry, a custom-made headplate (Protolabs) was cemented to the skull.

All implanted probes and headmounts were secured to the skull with three-part dental cement (Superbond, Sun Medical). Mice were allowed to recover from surgery for at least 2 wk before experiments.

### In Vivo Neural Recordings and Optogenetics.

Standard, dual-excitation-wavelength fiber photometry recordings and analyses [described in detail in previous work (16, 69, 70)] were used to obtain AGRP-GCaMP6s signals. HON-GCaMP6s population and individual cell activity (Figs. 4*A* and 5*B*) was recorded using an INSCOPIX head-mounted microscope, as described in detail in (16). Fluorescence outputs were converted to normalized photometry signal, % ΔF/F, as described in (70–72). Following the guidelines in (73), for between-subjects visualizations, the % ΔF/F data were then converted to z scores using the function z = (Fn – µ)/σ. Here, Fn is the normalized photometry signal, and µ and σ are the mean and SD (respectively) of Fn of the whole recording session, or of a local baseline relative to event or stimulus.

### Pupillometry and Data Integration.

Pupil dynamics were recorded in head-fixed mice using an infrared-sensitive camera (Blackfly S, FLIR Systems; Spinnaker SDK) at a frame rate of 20 to 30 Hz. To track pupil size, eight points around the pupil margin were identified in each video frame using DeepLabCut (74). Pupil radius was then calculated by applying a circle-fitting algorithm in MATLAB to these coordinates. Traces were preprocessed by interpolating blinks or low-confidence frames, and signals were low-pass filtered at 3 Hz.

For integration with calcium imaging data, photometry and pupil size data were aligned and downsampled to 10 Hz using the function resample, and z-scored across the whole trace. To visualize AN-GCaMP6s signals corresponding to similar pupil dilations in different mouse groups, dilation peaks were detected using findpeaks in Matlab (MinPeakProm = 1SD) and events in which running was less than 0.05 cm/s were removed. To analyze temporal correlations between AN-GCaMP6s signals and pupil size, AN events were detected using findpeaks in Matlab (min peak prominence 5% ΔF/F), time windows of 20 s around the peak of the event were taken, and cross-correlated with corresponding pupil data using the Matlab function xcorr with “coeff” normalization.

### In Vivo Optogenetics.

Optogenetic stimulation of HONs was performed as described in detail in our previous work (15). To quantify the magnitude of the optogenetic effect, we calculated the mean ΔF/F signal during the first 10 s of laser stimulation, subtracted by the mean baseline fluorescence acquired during the 20 s immediately preceding laser onset (Fig. 2*C*).

### Brain Slice Recordings and Optogenetics.

For brain slice VGAT-GCaMP recordings and optogenetics (Fig. 2 *D*–*F*), 300 μm hypothalamic coronal slices were acutely dissected and studied as described in detail in our previous work (75). The slices were placed in a recording chamber of an Olympus microscope, continuously perfused with ACSF (75) at 21 °C, gassed with 95% O2 and 5% CO2. To stimulate ChrimsonR expressed in HONs and axons, HEKA Patchmaster software was used to deliver 20 Hz, 30 s trains of 5 ms pulses of a red laser (635 nm, Laserglow Technologies, 10mW at the fiber tip) through a fiberoptic tip (200 μm diameter) placed above mediobasal or mediodorsal hypothalamic areas (defined coordinates below). Calcium signals were recorded using a UMPlanFL 10x objective, Olympus (0.3 NA,10 mm working distance) and Retiga ELECTRO camera (QImaging). Excitation light for calcium imaging was provided by a 1 Hz 465 nm Sutter Lambda 4DG light source, controlled by HEKA Patchmaster software. Images were acquired using HEKA SmartLUX and HEKA Patchmaster software. Regions of interest (ROIs) were manually labeled using ImageJ, and data were analyzed using MATLAB. Single-cell GCaMP signal was converted to z scores using mean and SD of its baseline (30 s before laser stimulation)

.

### Food Presentation Experiments.

Highly palatable food (HPF) was either milkshake or peanut butter (Fig. 4 *A* and *D* shows data from peanut butter, and Fig. 4 *B* and *C* from milkshake, but similar results were confirmed with both HPFs in all experiments). Animals were habituated to peanut butter (Migros Classic Crunchy, 6.04 kcal g^−1^) and milkshake (energy milk strawberry flavor, 0.76 kcal ml^−1^, Emmi AG) in home cages overnight and to experimental cages for a minimum of 2 d before experiments started. On experimental days, animals were placed in the experimental cage for 15 min, and then food was placed into the cage as described in (28). For pupil recording in the food-cue experiments, head-fixed animals were presented with the odor of peanut butter. A Falcon tube smeared with peanut butter was placed in line with a low-pressure air source, and the resulting odorized airstream was delivered through tubing gated by a solenoid valve, with the tube outlet positioned ~5 cm from the animal’s nose. The valve was opened five times for 5 s each, with a 60 s intertrial interval.

### Lickometer Analysis.

Palatable food consumption was measured using a custom-built HPF dispenser (cat. no. 161K011, NResearch) with a capacitor-based lick sensor (cat. no. AT42QT-1010, SparkFun Electronics); data were recorded at 500 Hz using custom Python scripts and a digital I/O device (NI-DAQmx, National Instruments). 6 µL of milkshake was dispensed for every 10 detected licks. In Fig. 5, lick data were z-scored to the local baseline period 5 s before the sound stimulus.

### Sudden Noise Experiments.

Mice were previously habituated to both the lickometer and milkshake. After mice licked continuously for 20 s, a white noise was played through a loudspeaker placed next to the lickometer (1 s duration, 120 dB), while licking and neural responses were recorded (Fig. 5 *B*–*E*). To quantify the AN response to the noise without the confound of licking and moving around the cage, AN responses were additionally recorded in the head-fixed setup (as used for pupillometry in Fig. 5*F*) without food. For pupil recordings, the animal was head-fixed on a running wheel and presented with a 1 s burst of white noise at 120 dB SPL (measured at the position of the animal’s head).

### Histology and Immunocytochemistry.

To validate the locations of AAV virus expression and the fiber optic, we perfused mice with PBS followed by buffered 4% paraformaldehyde (PFA, pH 7.4). Brains were then postfixed in 4% paraformaldehyde for 30 h before being placed in 30% sucrose solution for cryoprotection overnight. Brains were then sectioned on a Leica cryostat at 50 μm. For HON immunoreactivity, slices were incubated in primary antibody against hypocretin-A (Abcam ab6214) at a 1:250 dilution in 3% BSA/0.3% Triton solution overnight at 4 °C. Slices were then washed in PBS followed by incubation with AlexaFluor594 (at 1:1,000 dilution) at room temperature for 2 h. Slices were then washed in PBS. Images were acquired using a fluorescence microscope (Eclipse Ti2, Nikon).

### Data Analysis and Statistics.

Data processing and statistical evaluations were conducted using MATLAB (Mathworks, United States). All animals and data points were included in the analyses, except for those with no signal in fiber photometry at the beginning of experiments. The results of all statistical tests are summarized in figure legends (ns = not significant, *P < 0.05, **P < 0.01, ***P < 0.001). *t* tests were two-tailed, except where alternative hypotheses were directional in which case one-tailed tests were used. *P* values less than 0.05 were deemed statistically significant. Results and figures are displayed as data mean ± SEM, unless stated otherwise.

## Data Availability

All study data are included in the main text.

## References

[1] A. Engin, The definition and prevalence of obesity and metabolic syndrome. Adv. Exp. Med. Biol. **960**, 1–17 (2017). 10.1007/978-3-319-48382-5_1 28585193

[2] S. D. Anton , Flipping the metabolic switch: Understanding and applying the health benefits of fasting. Obesity **26**, 254–268 (2018). 10.1002/oby.22065 PMC5783752 29086496

[3] S. Golbidi , Health benefits of fasting and caloric restriction. Curr. Diab. Rep. **17**, 123 (2017). 10.1007/s11892-017-0951-7 29063418

[4] E. L. Lim , Reversal of type 2 diabetes: Normalisation of beta cell function in association with decreased pancreas and liver triacylglycerol. Diabetologia **54**, 2506–2514 (2011). 10.1007/s00125-011-2204-7 PMC3168743 21656330

[5] R. E. Patterson, D. D. Sears, Metabolic effects of intermittent fasting. Annu. Rev. Nutr. **37**, 371–393 (2017). 10.1146/annurev-nutr-071816-064634 PMC13170603 28715993

[6] C. Vernieri, F. Ligorio, D. Tripathy, V. D. Longo, Cyclic fasting-mimicking diet in cancer treatment: Preclinical and clinical evidence. Cell Metab. **36**, 1644–1667 (2024). 10.1016/j.cmet.2024.06.014 39059383

[7] L. Murta, D. Seixas, L. Harada, R. F. Damiano, M. Zanetti, Intermittent fasting as a potential therapeutic instrument for major depression disorder: A systematic review of clinical and preclinical studies. Int. J. Mol. Sci. **24**, 15551 (2023). 10.3390/ijms242115551 PMC10647529 37958535

[8] R. Fernandez-Rodriguez , Does intermittent fasting impact mental disorders? A systematic review with meta-analysis. Crit. Rev. Food Sci. Nutr. **63**, 11169–11184 (2023). 10.1080/10408398.2022.2088687 35713641

[9] B. Stapel , Impact of fasting on stress systems and depressive symptoms in patients with major depressive disorder: A cross-sectional study. Sci. Rep. **12**, 7642 (2022). 10.1038/s41598-022-11639-1 PMC9091273 35538177

[10] J. N. Betley , Neurons for hunger and thirst transmit a negative-valence teaching signal. Nature **521**, 180–185 (2015). 10.1038/nature14416 PMC4567040 25915020

[11] M. L. Andermann, B. B. Lowell, Toward a wiring diagram understanding of appetite control. Neuron **95**, 757–778 (2017). 10.1016/j.neuron.2017.06.014 PMC5657399 28817798

[12] M. J. Krashes , Rapid, reversible activation of AgRP neurons drives feeding behavior in mice. J. Clin. Invest. **121**, 1424–1428 (2011). 10.1172/JCI46229 PMC3069789 21364278

[13] S. N. Meissner , Self-regulating arousal via pupil-based biofeedback. Nat. Hum. Behav. **8**, 43–62 (2024). 10.1038/s41562-023-01729-z PMC10810759 37904022

[14] B. Y. Mileykovskiy, L. I. Kiyashchenko, J. M. Siegel, Behavioral correlates of activity in identified hypocretin/orexin neurons. Neuron **46**, 787–798 (2005). 10.1016/j.neuron.2005.04.035 PMC8281334 15924864

[15] N. Grujic, A. Tesmer, E. Bracey, D. Peleg-Raibstein, D. Burdakov, Control and coding of pupil size by hypothalamic orexin neurons. Nat. Neurosci. **26**, 1160–1164 (2023). 10.1038/s41593-023-01365-w PMC10322704 37336973

[16] M. M. Karnani , Role of spontaneous and sensory orexin network dynamics in rapid locomotion initiation. Prog. Neurobiol. **187**, 101771 (2020). 10.1016/j.pneurobio.2020.101771 PMC7086232 32058043

[17] M. van den Top, K. Lee, A. D. Whyment, A. M. Blanks, D. Spanswick, Orexigen-sensitive NPY/AgRP pacemaker neurons in the hypothalamic arcuate nucleus. Nat. Neurosci. **7**, 493–494 (2004). 10.1038/nn1226 15097991

[18] S. Muroya , Orexins (hypocretins) directly interact with neuropeptide Y, POMC and glucose-responsive neurons to regulate Ca 2+ signaling in a reciprocal manner to leptin: Orexigenic neuronal pathways in the mediobasal hypothalamus. Eur. J. Neurosci. **19**, 1524–1534 (2004). 10.1111/j.1460-9568.2004.03255.x 15066149

[19] B. Yang , Locus coeruleus anchors a trisynaptic circuit controlling fear-induced suppression of feeding. Neuron **109**, 823–838.e6 (2021). 10.1016/j.neuron.2020.12.023 PMC9272546 33476548

[20] I. de Araujo Salgado , Toggling between food-seeking and self-preservation behaviors via hypothalamic response networks. Neuron **111**, 2899–2917.e6 (2023). 10.1016/j.neuron.2023.06.006 PMC10528369 37442130

[21] X. Fang , Chronic unpredictable stress induces depression-related behaviors by suppressing AgRP neuron activity. Mol. Psychiatry **26**, 2299–2315 (2021). 10.1038/s41380-020-01004-x PMC8272726 33432188

[22] S. Mathot, Pupillometry: Psychology, physiology, and function. J. Cogn. **1**, 16 (2018). 10.5334/joc.18 PMC6634360 31517190

[23] N. Grujic, R. Polania, D. Burdakov, Neurobehavioral meaning of pupil size. Neuron **112**, 3381–3395 (2024). 10.1016/j.neuron.2024.05.029 38925124

[24] J. A. Gonzalez , Inhibitory interplay between orexin neurons and eating. Curr. Biol. **26**, 2486–2491 (2016). 10.1016/j.cub.2016.07.013 PMC5049542 27546579

[25] P. Viskaitis , Ingested non-essential amino acids recruit brain orexin cells to suppress eating in mice. Curr. Biol. **32**, 1812–1821.e4 (2022). 10.1016/j.cub.2022.02.067 35316652

[26] P. Viskaitis , Orexin neurons track temporal features of blood glucose in behaving mice. Nat. Neurosci. **27**, 1299–1308 (2024). 10.1038/s41593-024-01648-w PMC11239495 38773350

[27] Q. Tong, C. P. Ye, J. E. Jones, J. K. Elmquist, B. B. Lowell, Synaptic release of GABA by AgRP neurons is required for normal regulation of energy balance. Nat. Neurosci. **11**, 998–1000 (2008). 10.1038/nn.2167 PMC2662585 19160495

[28] Y. Chen, Y. C. Lin, T. W. Kuo, Z. A. Knight, Sensory detection of food rapidly modulates arcuate feeding circuits. Cell **160**, 829–841 (2015). 10.1016/j.cell.2015.01.033 PMC4373539 25703096

[29] L. Duffet , A genetically encoded sensor for in vivo imaging of orexin neuropeptides. Nat. Methods **19**, 231–241 (2022). 10.1038/s41592-021-01390-2 PMC8831244 35145320

[30] Y. Li, A. N. van den Pol, Differential target-dependent actions of coexpressed inhibitory dynorphin and excitatory hypocretin/orexin neuropeptides. J. Neurosci. **26**, 13037–13047 (2006). 10.1523/JNEUROSCI.3380-06.2006 PMC6674960 17167093

[31] T. C. Chou , Orexin (hypocretin) neurons contain dynorphin. J. Neurosci. **21**, RC168 (2001). 10.1523/JNEUROSCI.21-19-j0003.2001 PMC6762880 11567079

[32] C. Schöne, J. Apergis-Schoute, T. Sakurai, A. Adamantidis, D. Burdakov, Coreleased orexin and glutamate evoke nonredundant spike outputs and computations in histamine neurons. Cell Rep. **7**, 697–704 (2014). 10.1016/j.celrep.2014.03.055 PMC4022832 24767990

[33] C. S. Thomas , Optogenetic stimulation of lateral hypothalamic orexin/dynorphin inputs in the ventral tegmental area potentiates mesolimbic dopamine neurotransmission and promotes reward-seeking behaviours. Neuropsychopharmacology **47**, 728–740 (2022). 10.1038/s41386-021-01196-y PMC8782948 34663867

[34] X. Yu , Wakefulness is governed by GABA and histamine cotransmission. Neuron **87**, 164–178 (2015). 10.1016/j.neuron.2015.06.003 PMC4509551 26094607

[35] D. Wang , Whole-brain mapping of the direct inputs and axonal projections of POMC and AgRP neurons. Front. Neuroanat. **9**, 40 (2015). 10.3389/fnana.2015.00040 PMC4375998 25870542

[36] Y. Mandelblat-Cerf , Arcuate hypothalamic AgRP and putative POMC neurons show opposite changes in spiking across multiple timescales. elife **4**, e07122 (2015). 10.7554/eLife.07122 PMC4498165 26159614

[37] L. R. Beutler , Dynamics of gut-brain communication underlying hunger. Neuron **96**, 461–475.e5 (2017). 10.1016/j.neuron.2017.09.043 PMC5691364 29024666

[38] Z. Su, A. L. Alhadeff, J. N. Betley, Nutritive, post-ingestive signals are the primary regulators of AgRP neuron activity. Cell Rep. **21**, 2724–2736 (2017). 10.1016/j.celrep.2017.11.036 PMC5724395 29212021

[39] A. Gouveia, R. de Oliveira Beleza, S. M. Steculorum, AgRP neuronal activity across feeding-related behaviours. Eur. J. Neurosci. **54**, 7458–7475 (2021). 10.1111/ejn.15498 34655481

[40] O. K. Hassani, M. R. Krause, L. Mainville, C. A. Cordova, B. E. Jones, Orexin neurons respond differentially to auditory cues associated with appetitive versus aversive outcomes. J. Neurosci. **36**, 1747–1757 (2016). 10.1523/JNEUROSCI.3903-15.2016 PMC4737782 26843654

[41] L. van der Post, Quote attributed to Kalahari bushmen as documented by Sir Laurence van der Post; taken from the article “Hunter-Gathers” by Simon Belcher. https://royalafricansafaris.com/articles/hunter-gatherers/ (Accessed 29 april 2026).

[42] A. K. Sutton Hickey, M. J. Krashes, Integrating hunger with rival motivations. Trends Endocrinol. Metab. **31**, 495–507 (2020). 10.1016/j.tem.2020.04.006 32387196

[43] A. L. Alhadeff , A neural circuit for the suppression of pain by a competing need state. Cell **173**, 140–152.e5 (2018). 10.1016/j.cell.2018.02.057 PMC5877408 29570993

[44] C. J. Burnett , Hunger-driven motivational state competition. Neuron **92**, 187–201 (2016). 10.1016/j.neuron.2016.08.032 PMC5082717 27693254

[45] I. Pavlov, Conditioned reflexes (OUP, 1927).

[46] E. N. Sokolov, Higher nervous functions; The orienting reflex. Annu. Rev. Physiol. **25**, 545–580 (1963). 10.1146/annurev.ph.25.030163.002553 13977960

[47] I. M. Sechenov, Reflexes of the Brain, The MIT Paperback Series (M.I.T. Press, Cambridge, 1965), p. 149.

[48] J. D. Cohen, S. M. McClure, A. J. Yu, Should I stay or should I go? How the human brain manages the trade-off between exploitation and exploration. Philos. Trans. R. Soc. Lond. B. Biol. Sci. **362**, 933–942 (2007). 10.1098/rstb.2007.2098 PMC2430007 17395573

[49] T. L. Horvath, S. Diano, A. N. van den Pol, Synaptic interaction between hypocretin (orexin) and neuropeptide Y cells in the rodent and primate hypothalamus: A novel circuit implicated in metabolic and endocrine regulations. J. Neurosci. **19**, 1072–1087 (1999). 10.1523/JNEUROSCI.19-03-01072.1999 PMC6782143 9920670

[50] S. Kosar, E. Bracey, Y. Du, D. Peleg-Raibstein, D. Burdakov, A neural circuit for rapid control of hunger by arousal. bioRxiv [Preprint] (2025). 10.1101/2025.03.07.642010.

[51] A. Malcolm, W. L. Toh, K. Crocker, A. Phillipou, The contribution of sleep to anorexia nervosa severity. Eat. Weight Disord. **27**, 1563–1568 (2022). 10.1007/s40519-021-01286-2 34426951

[52] P. Burger, R. W. Bos, J. Maas, M. Simeunovic-Ostojic, R. Gemke, Sleep disturbances in anorexia nervosa. Eur. Eat. Disord. Rev. **33**, 318–342 (2025). 10.1002/erv.3148 PMC11786938 39444255

[53] V. Malafarina, F. Uriz-Otano, L. Gil-Guerrero, R. Iniesta, The anorexia of ageing: Physiopathology, prevalence, associated comorbidity and mortality. A systematic review. Maturitas **74**, 293–302 (2013). 10.1016/j.maturitas.2013.01.016 23415063

[54] B. A. Mander, J. R. Winer, M. P. Walker, Sleep and human aging. Neuron **94**, 19–36 (2017). 10.1016/j.neuron.2017.02.004 PMC5810920 28384471

[55] K. K. Gulia, V. M. Kumar, Sleep disorders in the elderly: A growing challenge. Psychogeriatrics **18**, 155–165 (2018). 10.1111/psyg.12319 29878472

[56] S. B. Li , Hyperexcitable arousal circuits drive sleep instability during aging. Science **375**, eabh3021 (2022). 10.1126/science.abh3021 PMC9107327 35201886

[57] V. Baldini , Eating disorders in narcolepsy type 1: Evidence from a cross-sectional Italian study. J. Sleep Res. **33**, e14150 (2024). 10.1111/jsr.14150 38351712

[58] H. A. Fortuyn , High prevalence of eating disorders in narcolepsy with cataplexy: A case-control study. Sleep **31**, 335–341 (2008). 10.1093/sleep/31.3.335 PMC2276738 18363309

[59] R. J. van Holst , Aberrant food choices after satiation in human orexin-deficient narcolepsy type 1. Sleep **39**, 1951–1959 (2016). 10.5665/sleep.6222 PMC5070749 27568806

[60] E. Akcay , Pupillary dilation response to the auditory food words in adolescents with obesity without binge eating disorder. Biol. Psychol. **193**, 108874 (2024). 10.1016/j.biopsycho.2024.108874 39313180

[61] R. Graham, A. Hoover, N. A. Ceballos, O. Komogortsev, Body mass index moderates gaze orienting biases and pupil diameter to high and low calorie food images. Appetite **56**, 577–586 (2011). 10.1016/j.appet.2011.01.029 21291928

[62] C. Li , AGRP neurons modulate fasting-induced anxiolytic effects. Transl. Psychiatry **9**, 111 (2019). 10.1038/s41398-019-0438-1 PMC6408535 30850579

[63] W. Chen , Nutrient-sensing AgRP neurons relay control of liver autophagy during energy deprivation. Cell Metab. **35**, 786–806.e3 (2023). 10.1016/j.cmet.2023.03.019 PMC10173804 37075752

[64] M. L. Klima , Anti-inflammatory effects of hunger are transmitted to the periphery via projection-specific AgRP circuits. Cell Rep. **42**, 113338 (2023). 10.1016/j.celrep.2023.113338 PMC10749715 37910501

[65] J. P. Cavalcanti de Albuquerque , Brain sensing of metabolic state regulates circulating monocytes. Sci. Immunol. **10**, eadr3226 (2025). 10.1126/sciimmunol.adr3226 40184437

[66] A. S. Garfield , Dynamic GABAergic afferent modulation of AgRP neurons. Nat. Neurosci. **19**, 1628–1635 (2016). 10.1038/nn.4392 PMC5382799 27643429

[67] J. Berrios , Food cue regulation of AGRP hunger neurons guides learning. Nature **595**, 695–700 (2021). 10.1038/s41586-021-03729-3 PMC8522184 34262177

[68] N. Sayar-Atasoy , AgRP neurons encode circadian feeding time. Nat. Neurosci. **27**, 102–115 (2024). 10.1038/s41593-023-01482-6 PMC11657780 37957320

[69] A. L. Tesmer , Orexin neurons mediate temptation-resistant voluntary exercise. Nat. Neurosci. **27**, 1774–1782 (2024). 10.1038/s41593-024-01696-2 PMC11374669 39107488

[70] T. N. Lerner , Intact-brain analyses reveal distinct information carried by SNc dopamine subcircuits. Cell **162**, 635–647 (2015). 10.1016/j.cell.2015.07.014 PMC4790813 26232229

[71] C. K. Kim , Simultaneous fast measurement of circuit dynamics at multiple sites across the mammalian brain. Nat. Methods **13**, 325–328 (2016). 10.1038/nmeth.3770 PMC5717315 26878381

[72] C. Concetti, E. F. Bracey, D. Peleg-Raibstein, D. Burdakov, Control of fear extinction by hypothalamic melanin-concentrating hormone-expressing neurons. Proc. Natl. Acad. Sci. U.S.A. **117**, 22514–22521 (2020). 10.1073/pnas.2007993117 PMC7486764 32848057

[73] V. N. Sherathiya, M. D. Schaid, J. L. Seiler, G. C. Lopez, T. N. Lerner, GuPPy, a Python toolbox for the analysis of fiber photometry data. Sci. Rep. **11**, 24212 (2021). 10.1038/s41598-021-03626-9 PMC8688475 34930955

[74] A. Mathis , DeepLabCut: Markerless pose estimation of user-defined body parts with deep learning. Nat. Neurosci. **21**, 1281–1289 (2018). 10.1038/s41593-018-0209-y 30127430

[75] D. Burdakov, M. M. Karnani, Ultra-sparse connectivity within the lateral hypothalamus. Curr. Biol. **30**, 4063–4070.e2 (2020). 10.1016/j.cub.2020.07.061 PMC7575142 32822604

